# Management of Spontaneously Ruptured Hepatocellular Carcinomas in the Radiofrequency Ablation Era

**DOI:** 10.1371/journal.pone.0094453

**Published:** 2014-04-09

**Authors:** Tan To Cheung, Ronnie T. P. Poon, Kenneth S. H. Chok, Albert C. Y. Chan, Simon H. Y. Tsang, Wing Chiu Dai, Thomas C. C. Yau, See Ching Chan, Sheung Tat Fan, Chung Mau Lo

**Affiliations:** Department of Surgery, Queen Mary Hospital, The University of Hong Kong, Hong Kong, China; The First Affiliated Hospital of Nanjing Medical University, China

## Abstract

**Background and aim:**

Spontaneous rupture of hepatocellular carcinoma (HCC) carries a high mortality. The use of radiofrequency ablation (RFA) in recent years has enriched the armamentarium for hemostasis of spontaneously ruptured HCCs but its results have not been documented. This study investigated the prognosis and outcome of spontaneous rupture of HCC as well as the results of using RFA for hemostasis.

**Patients and method:**

From January 1991 to December 2010, 5283 patients were diagnosed with HCC at our hospital, and 189 of them had spontaneous rupture of HCCs. They were grouped under two periods: period 1, 1991–2000, n = 70; period 2, 2001–2010, n = 119. RFA was available in period 2 only.

**Results:**

Hepatitis B virus infection was predominant in both periods. Surgical hemostasis was mainly achieved by hepatic artery ligation in period 1 and by RFA in period 2. The 30-day hospital mortality after surgical treatment was 55.6% (n = 18) in period 1 and 19.2% (n = 26) in period 2 (p = 0.012). Multivariate analysis identified 4 independent factors for better overall survival, namely, hemostasis by transarterial chemoembolization (hazard ratio 0.516, 95% confidence interval 0.354–0.751), hemostasis by RFA (hazard ratio 0.431, 95% confidence interval 0.236–0.790), having surgery as a subsequent treatment (hazard ratio 0.305, 95% confidence interval 0.186–0.498), and a serum total bilirubin level <19 umol/L (hazard ratio 1.596, 95% confidence interval 1.137–2.241).

**Conclusion:**

The use of RFA for hemostasis during laparotomy greatly reduced the hospital mortality rate when compared with conventional hepatic artery ligation.

## Introduction

Spontaneous rupture is one of the presentations of hepatocellular carcinoma (HCC). It can have catastrophic consequences. The incidence of spontaneous rupture of HCCs ranges from 3–14.5% in regions where HCC is prevalent [1]–[4]. Transarterial chemoembolization (TACE) is a well-established treatment option [5], [6]. Emergency hepatic artery ligation, plication, packing, hepatectomy or radiofrequency ablation (RFA) is required if TACE fails to achieve hemostasis [3], [4]. Unfortunately, many patients having surgical intervention end up in liver failure or even death. This study tried to find out whether using RFA for hemostasis during laparotomy would improve patients' hospital survival.

## Patients and Methods

From January 1991 to December 2010, 5283 patients were diagnosed with HCC and 189 of them had spontaneous rupture of HCCs and were admitted to our hospital. All the data used in this study were recorded by a single research assistant. The 189 patients were grouped under two periods: period 1, 1991–2000, n = 70; period 2, 2001–2010, n = 119.

### Ethics statement

The study complies with the laws of Hong Kong and follows the principles laid down in the Declaration of Helsinki. Oral consent by the patients was obtained after clear and complete explanation and was recorded in the patients' medical records. Approval in the form of written consent was not needed as it was waived by the institutional review board of The University of Hong Kong, which approved this retrospective study.

### Treatment algorithm

Diagnosis of spontaneous rupture of HCCs was based on clinical pictures acquired by physical examination and screening ultrasonography on presentation. Shock index was used to describe the hemodynamic status of a patient. Shock index was calculated as the division of heart rate reading by systolic blood pressure reading. The shock index of a healthy adult should be in the range between 0.5 and 0.7. A patient was classified as in shock when his/her shock index was >0.7 [7].

**Figure 1:**
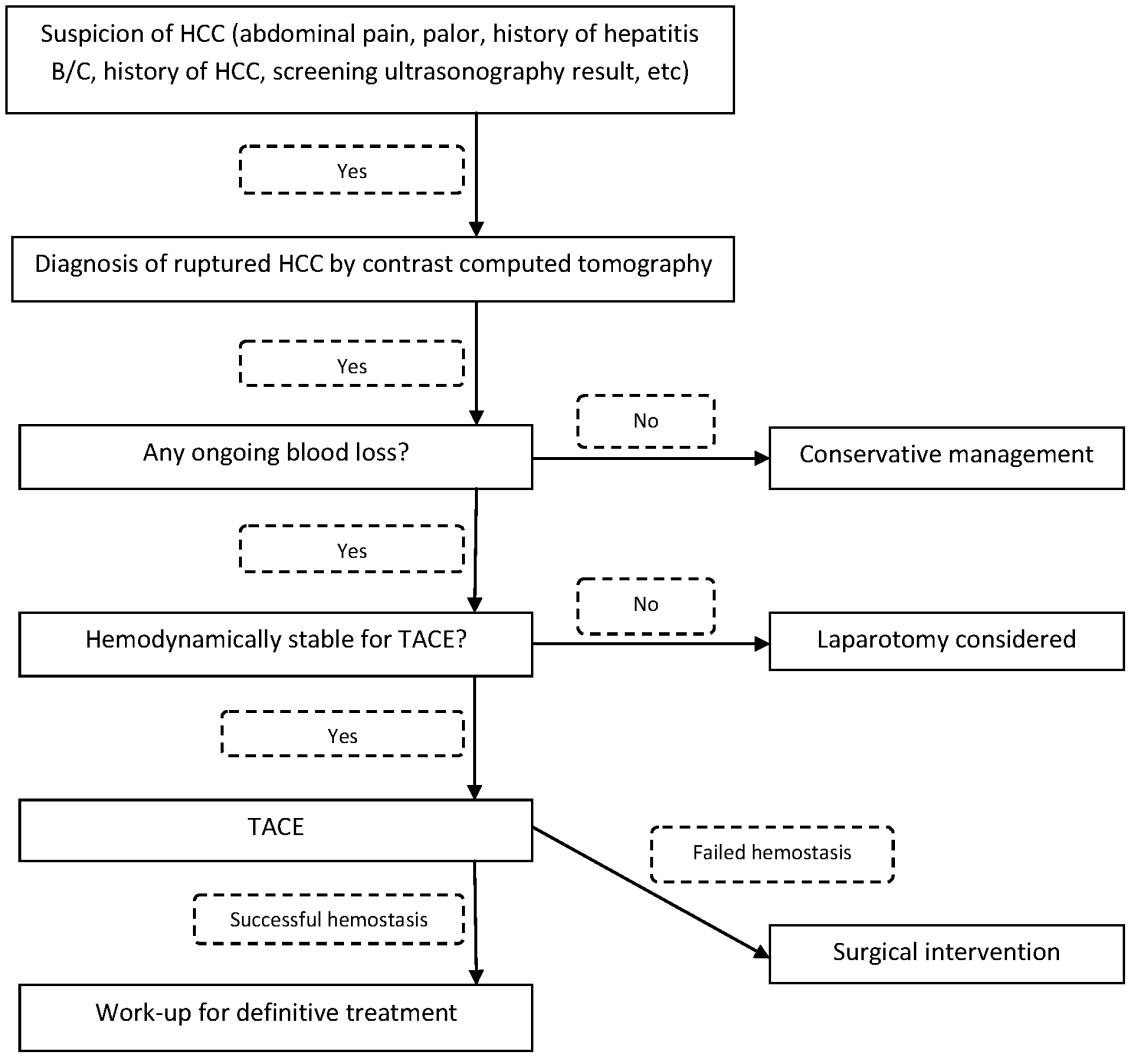
Treatment algorithm for spontaneous rupture of HCCs.

Contrast computed tomography of the abdomen was performed for patients without renal impairment. Patients with any one of the following three conditions were managed conservatively: (a) major portal vein tumor thrombosis, (b) disseminated extrahepatic disease, (c) Karnosfsky performance status <40%. Other patients were treated according to our standard treatment protocol (Figure 1).

### TACE

TACE was conducted by experienced interventional radiologists. Selective cannulation of the feeding artery of the tumor was attempted. Embolization of the feeding artery was performed by injection of pellets (1×2 mm) mixed with 40 mg of gentamycin and contrast. Depending on the size and number of tumor, 4–20 mL of gelfoam emulsion was injected into the catheter placed in the artery supplying the tumor or, for bilobar disease, into the hepatic artery proper beyond the gastroduodenal artery. Gelfoam injection was stopped when the blood flow in the artery supplying the tumor slowed down and occlusion occurred. Relative contraindications to TACE included main portal vein thrombosis, arteriovenous shunting and Child-Pugh C cirrhosis [8].

### Operative treatment

All operations were conducted by experienced hepatobiliary surgeons. A bilateral subcostal incision with an upward midline incision was performed in most of the patients. Abdominal packing was performed. Depending on the size of the ruptured tumor and the surgeon's clinical judgement [4], [9], [10], hemostasis was attempted by (a) direct suture of the bleeding site, (b) selective hepatic artery ligation, (c) ligation of the common trunk of the hepatic artery, (d) hepatectomy, or (e) RFA (available in period 2 only). If hepatectomy was required, hepatic parenchymal transection was conducted with an ultrasonic dissector. The Pringle maneuver might be applied during the procedure. All patients who received operative treatment were managed in the intensive care unit.

### RFA

RFA was available in period 2 only. It was performed by hepatobiliary surgeons experienced in using the RFA device. Direct puncturing of the bleeding site was performed in most of the cases. A single needle or clustered needles (maximum array diameter 4.0 cm) were used according to the size of the tumor. For most bleeding tumors, a cluster probe was used. RFA was administered using the Olympus Cool-tip system, which consists of a generator that supplies up to 200 W of power until satisfactory hemostasis is achieved [11], [12].

### Statistical analysis

The baseline characteristics of patients were expressed as medians and ranges. The Mann-Whitney U test was used to compare continuous variables, and Pearson's chi-squared test was used to compare discrete variables. Hospital mortality was defined as death occurring after admission to and before discharge from hospital. Survival curves were computed with the Kaplan-Meier method and compared between groups by the log-rank test. The logistic regression analysis was performed to define factors that affected hospital mortality. The Cox proportional hazards model was used to define factors that determined the 30-day mortality rate. Statistical significance was denoted by a p value <0.05. All statistical calculations used the computer software SPSS/PC+ (SPSS, Chicago, IL, USA).

## Results

The rate of spontaneous rupture of HCC in period 1 was 3.7% (70/1892) and 3.5% (119/3391) in period 2. Demographics, liver function and clinical presentations of the 189 patients are shown in Table 1. In period 1, 33 (47.1%) patients received TACE as the first treatment, 15 (21.4%) patients received surgery as the first treatment, and 22 (31.4%) patients received conservative management. Hemostasis by TACE failed in 3 patients and they subsequently received surgery. In period 2, 58 (48.7%) patients received TACE as the first treatment, 20 (16.8%) patients received surgery as the first treatment, and 41 (34.5%) patients received conservative management. Hemostasis by TACE failed in 6 patients and they subsequently received surgery.

**Table 1:** Demographics, liver function and clinical presentations of patients.

	1991–2000 (n = 70)	2001–2010 (n = 119)	P
Median age (years)	59.5 (13–94)	58 (34–92)	0.557
Male: Female	57∶13	88∶31	0.240
Presence of comorbidity	23 (32.9%)	48 (40.3%)	0.305
Child-Pugh class			0.997
A	30 (46.2%)	55 (46.2%)	
B	26 (40.0%)	48 (40.3%)	
C	9 (13.8%)	16 (13.4%)	
Median Model for End-stage Liver Disease score	12.75 (6–27)	10.94 (6–37)	0.522
Median albumin (g/L)	32 (13–52)	34 (12–46)	0.332
Median total bilirubin (umol/L)	21 (6–750)	18 (4–180)	0.272
Median international normalized ratio	1.2 (0.9–3.2)	1.15 (0.9–4.1)	0.514
Median aspartate transaminase (U/L)	103 (23–1710)	93 (21–1003)	0.946
Median alanine transaminase (U/L)	54.5 (13–652)	50 (5–679)	0.221
Median creatinine (umol/L)	94 (46–379)	93 (8–296)	0.328
Median α-fetoprotein (ng/mL)	2055.5 (2–1720600)	636.5 (2–1451000)	0.236
Median tumor size (cm)	10 (2–23)	8.5 (2–30)	0.619
Hepatitis B virus infection	42 (60.0%)	86 (72.9%)	0.001
Peritoneal irritation or severe abdominal pain	49 (70.0%)	93 (78.2%)	0.211
Shock	33 (47.1%)	51 (42.9%)	0.674
Drop in hemoglobin >2 g/dL or hemoglobin <10 g/dL on initial blood test	46 (65.7%)	81 (68.1%)	0.739

In period 1, the rate of successful hemostasis was 78.8% (26 patients) for TACE, 77.8% (14 patients) for surgery, and 63.6% (14 patients) for conservative management. In period 2, the rate of successful hemostasis was 75.9% (44 patients) for TACE, 92.3% (24 patients) for surgery, and 58.5% (24 patients) for conservative management. In period 2, surgical hemostasis was mainly achieved by RFA. Operative and postoperative details and mortality rates in the two periods are shown in Table 2.

**Table 2:** Operative and postoperative details and mortality rates.

	1991–2000 (n = 18)	2001–2010 (n = 26)	P
Laparotomy without TACE	15 (83.3%)	20 (76.9%)	0.890
Common hepatic artery ligation	1 (5.6%)	0	0.852
Left or right hepatic artery ligation	12 (66.7%)	3 (11.5%)	0.0001
Direct suture	3 (16.7%)	1 (3.8%)	0.357
Tumor resection	2 (11.1%)	2 (7.7%)	1
Coagulation by argon beam	6 (33.3%)	12 (46.2%)	0.395
Hemostasis by RFA	0	19 (73.1%)	<0.0001
Median blood loss (L)	3 (0.8–13)	2.2 (0.2–7.8)	0.355
Median operation time (min)	115 (75–225)	140 (90–255)	0.102
Median intensive care unit stay (d)	2 (0–16)	2 (0–25)	0.761
Median hospital stay (d)	13 (1–34)	14.5 (0.46–52)	0.872
Complication (Clavien-Dindo grade)			
3a	2 (11.1%)	3 (11.5%)	1
3b	0	1 (3.8%)	1
4a	1 (5.6%)	1 (3.8%)	1
5	10 (55.6%)	6 (23.1%)	0.028
Mortality for hepatic artery ligation	8/13 (61.5%)	2/3 (66.7%)	1
Hospital mortality for ruptured HCC	10 (55.6%)	6 (23.1%)	0.028
30-day mortality for ruptured HCC	10 (55.6%)	5 (19.2%)	0.012

In period 2, 19 patients received RFA for hemostasis (Table 3). They had a median tumor size of 8.25 cm (range 3–19 cm). Eight (42.1%) patients had 1 tumor, 4 (21.1%) patients had 2 tumors, and 7 (36.8%) patients had >2 tumors. Single-needle probe was applied on 2 (10.5%) occasions and cluster-needle probe was applied on 17 (89.5%) occasions. The median ablation time was 22 min (range 12–72 min). Only 1 patient, who had a 10-cm tumor, received the Pringle maneuver during ablation, and the total inflow occlusion time was 60 min. Six (31.6%) patients who received RFA for hemostasis were found to have complete tumor ablation on computed tomography one month after the ablation.

**Table 3:** Details of RFA for hemostasis.

	Tumor <5 cm (n = 4)	Tumor 5–10 cm (n = 8)	Tumor >10 cm (n = 7)	P
Median tumor size (cm)	3.5 (3.0–4.5)	5.6 (5.0–9.1)	13.5 (11.1–19.0)	0.001
Tumor number				0.253
1	3 (75%)	2 (25.0%)	3 (42.9%)	
2	1 (25%)	3 (37.5%)	0	
>2	0	3 (37.5%)	4 (57.1%)	
Median ablation time (min)	22 (12–22)	17 (12–49)	32 (12–72)	0.222
No. of patients who had the Pringle maneuver	0	1 (12.5%)	0	
Time of the Pringle maneuver (min)	-	60	-	

**Figure 2:**
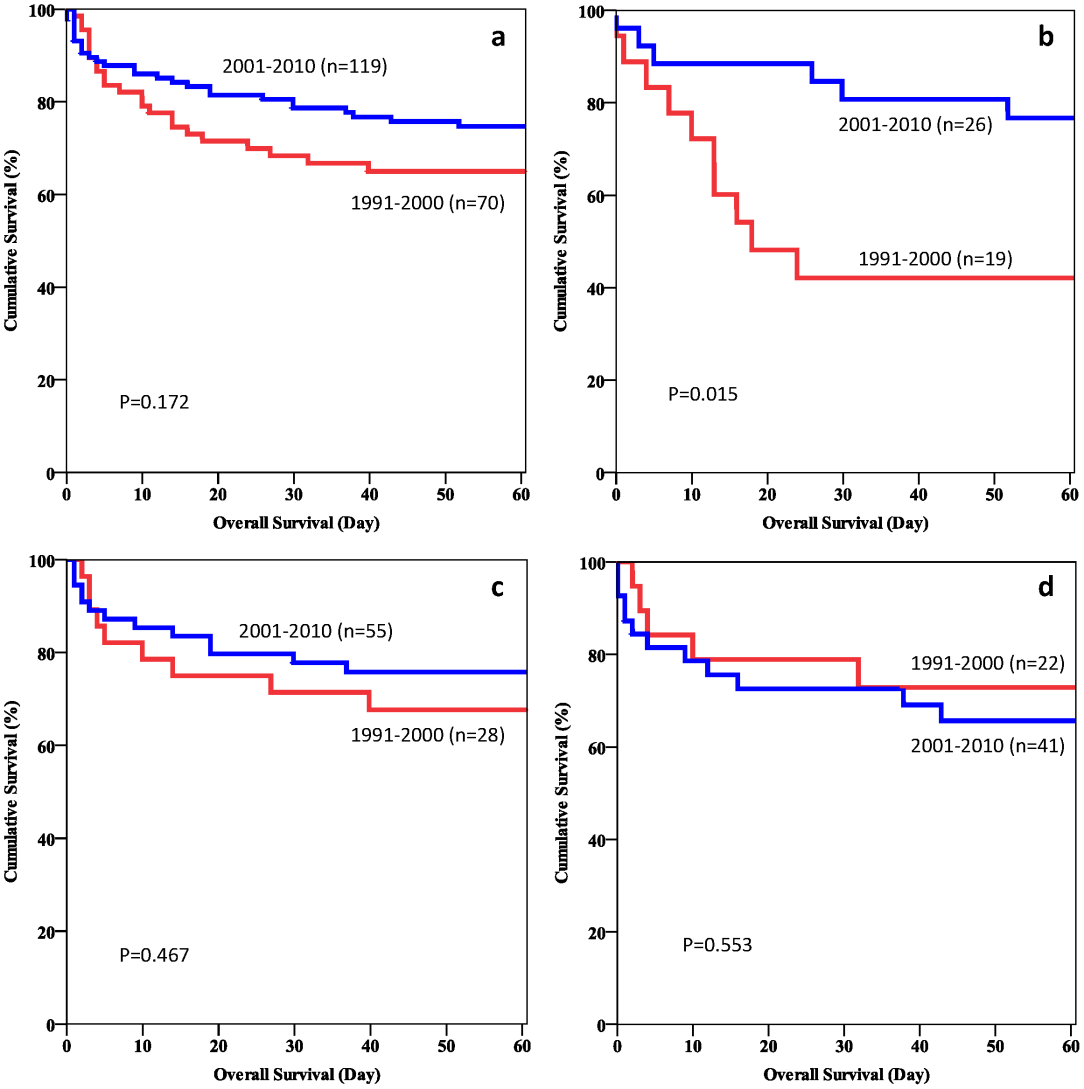
a. Overall hospital mortality of patients with spontaneously ruptured HCCs. b. Hospital mortality of patients who had surgery for spontaneously ruptured HCCs. c. Hospital mortality of patients who had TACE as the primary treatment for spontaneously ruptured HCCs. d. Hospital mortality of patients who had conservative management alone for spontaneously ruptured HCCs.

The 30-day hospital mortality rate of patients who had surgery was 55.6% in period 1 and 19.2% in period 2 (p = 0.012). The 30-day hospital mortality rate of patients who had TACE as the first treatment was 11.5% in period 1 and 6.8% in period 2 (p = 0.81). The 30-day hospital mortality rate of patients who had conservative management was 18.2% in period 1 and 24.4% in period 2 (p = 0.805). Figures 2a-2d show the 60-day survival curves of different groups of patients in the two periods.

In period 1, 11 patients had tumor resection after spontaneous rupture of tumors. Five of them had minor hepatectomy and 6 had major hepatectomy (resection of >3 liver segments). In period 2, 14 patients had tumor resection after spontaneous rupture of tumors. Four of them had minor hepatectomy and 10 had major hepatectomy. In period 2, 11 patients had RFA and 1 patient had high-intensity focused ultrasound ablation [13], [14] for their HCCs. One patient had liver transplantation at another hospital.

**Figure 3:**
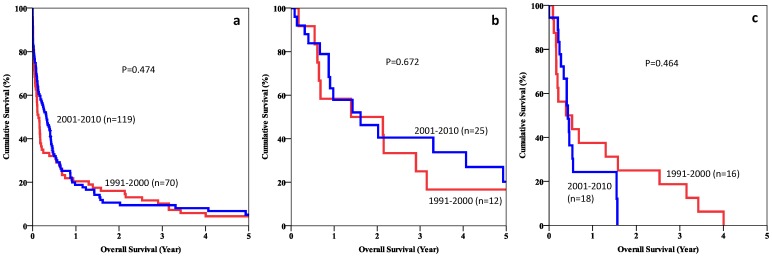
a. Overall survival of patients with spontaneously ruptured HCCs. b. Overall survival of patients who had hepatectomy or RFA for spontaneously ruptured HCCs. c. Overall survival of patients who had TACE for spontaneously ruptured HCCs.

The median survival of patients with spontaneous rupture of HCCs was 1.61 months (range 0–178 months) in period 1 and 3.84 months (range 0–118 months) in period 2 (p = 0.474). Sixteen patients in period 1 and 19 patients in period 2 received TACE as a subsequent treatment. Figures 3a–3c show the 5-year survival curves of different groups of patients in the two periods.

Thirteen independent factors that might affect the survival outcome were analyzed (Table 4). The 6 independent risk factors for poor survival were (a) tumor size >10 cm, (b) intolerance of TACE for hemostasis, (c) unsuitability for subsequent surgery, (d) poor Child-Pugh grade, (e) serum total bilirubin level >19 umol/L, and (f) international normalized ratio >1.2.

**Table 4:** Factors that might affect patient survival.

	1991–2000			2001–2010			1991–2010		
	No. of patients	Median survival (months)	P	No. of patients	Median survival (months)	P	No. of patients	Median survival (months)	P
Tumor size (cm)			0.003			0.001			<0.0001
≤10	31	4.63		62	5.06		93	5.06	
>10	34	1.25		56	1.41		90	1.35	
Hemostasis by RFA			-			0.089			0.083
No	70	1.61		100	3.16		170	2.10	
Yes	0	-		19	6.61		19	6.61	
Hemostasis by surgery			0.588			0.870			0.653
No	52	1.84		106	3.68		158	2.33	
Yes	18	0.59		13	3.88		31	3.29	
Hemostasis by TACE			0.003			0.133			0.001
No	44	1.08		70	2.89		114	1.71	
Yes	26	6.54		49	4.86		75	5.06	
Subsequent TACE			0.068			0.517			0.120
Yes	16	4.63		19	5.16		35	5.16	
No	54	1.05		100	3.02		154	1.61	
Subsequent surgery			0.001			<0.0001			<0.0001
Yes	12	16.69		16	17.12		28	19.45	
No	58	1.22		103	2.56		161	1.61	
Shock on presentation			0.097			0.149			0.96
No	37	2.14		68	3.68		105	3.02	
Yes	33	0.59		51	4.83		84	1.38	
Child-Pugh class			<0.0001			0.197			<0.0001
A	30	8.18		55	4.86		85	5.75	
B	26	0.79		48	2.76		74	1.25	
C	9	0.36		16	0.62		25	0.62	
MELD score			0.0004			0.399			0.123
≤11	28	7.76		61	3.85		89	4.04	
>11	33	1.22		57	2.76		90	1.31	
Total bilirubin (umol/L)			0.0002			0.001			<0.0001
≤19	32	6.54		62	5.52		94	5.75	
>19	36	1.05		57	1.35		93	1.22	
Platelet (×10^9^/L)			0.679			0.168			0.449
≤192.5	41	1.35		53	5.06		94	3.85	
>192.5	27	1.94		66	2.56		93	2.17	
International normalized ratio			0.001			0.621			0.019
≤1.2	37	2.53		77	4.47		114	4.04	
>1.2	25	0.89		41	1.51		66	1.22	
Albumin (g/L)			0.063			0.443			0.086
≤34	35	1.08		67	2.89		102	1.31	
>34	33	4.63		52	4.24		85	4.47	

MELD score, Model for End-stage Liver Disease score (to the nearest integer of the median).

On multivariate analysis by the Cox regression model, 4 independent factors for improved survival were identified (Table 5). They were (a) hemostasis by TACE, (b) hemostasis by RFA, (c) having surgery as a subsequent treatment, and (d) a serum total bilirubin level <19 umol/L.

**Table 5:** Factors for improved patient survival.

	P	Hazard ratio	95% confidence interval
Hemostasis by TACE	0.0006	0.516	0.354–0.751
Hemostasis by RFA	0.0064	0.431	0.236–0.790
Total bilirubin >19 umol/L	0.0069	1.596	1.137–2.241
Surgery as a subsequent treatment	<0.0001	0.305	0.186–0.498

## Discussion

To our knowledge, this study has the largest series of patients with cirrhosis and spontaneously ruptured HCCs. Spontaneous rupture is not the most common clinical presentation of HCCs but it can lead to catastrophic consequences. In the study, 3.7% and 3.5% of the HCC patients in period 1 and period 2 respectively had spontaneous rupture of tumors. Compared with the historic figure of 14.5% published by Ong et al. [9], there was a drastic reduction of this clinical manifestation. Early screening of patients with hepatitis B virus infection might have allowed detection of HCC at an early stage. The use of more sophisticated imaging modality like dual-tracer positron emission tomography/computed tomography could have subjected more patients to surgery when the tumors were still small [15], [16].

The management of spontaneously ruptured HCCs remains difficult because patients often present with very poor physical conditions including coagulopathy and hemodynamic instability [17], [18]. A clear and comprehensive treatment protocol for these scenarios is essential. TACE remains as the first-line hemostatic option for patients with a relatively stable hemodynamic condition [19]–[21]. TACE is considered highly effective in hemostasis for ruptured HCCs. In the study, the rates of successful hemostasis in the two periods were comparable. However, the ischemic effect of TACE on cirrhotic livers might have contributed to liver failure, leading to a hospital mortality of 20–30% in the entire period.

In period 1, the hospital mortality after surgery was substantial; more than half of the patients did not survive. On the contrary, there was a significant improvement in hospital mortality after surgery in period 2. The operation mortality reduced to 23%. In period 1, one of the surgical strategies for hemostasis was direct suture of the tumour [4]. This method, however, was far from satisfactory. Since the nature of liver tumors is quite soft, many of the sutures might have cut through the tumors, ending up in ineffective hemostasis. In spontaneous rupture of HCCs, the bleeding mainly comes from the hepatic artery [22]. Selective hepatic artery ligation was applied in period 1. Ligation of the common hepatic artery was performed for patients with ruptured tumors involving both lobes of the liver if selective ligation of the hepatic artery branch had failed to stop the bleeding. Nevertheless, successful cessation of bleeding by hepatic artery ligation could not secure successful survival. More than 60% of the patients ended up in hospital mortality secondary to liver failure. Neither selective ligation of hepatic artery nor ligation of the common hepatic artery was well tolerated in patients with advanced cirrhosis and HCC. In period 2, selective or non-selective hepatic artery ligation also resulted in poor survival outcomes. Three patients who had very unstable hemodynamic condition during operation had ligation of the hepatic artery as a last resort. Two patients developed liver failure as a complication of hepatic artery ligation.

RFA was initially invented as an ablative tool for small HCCs in an elective setting [23]–[25]. It played an important role in period 2 as a treatment for spontaneous rupture of HCCs. RFA can achieve hemostasis and complete ablation at the same time for patients with ruptured HCCs. For lesions <3 cm, a single probe can effectively achieve complete ablation [22], [25], [26]. A cluster probe has an effective ablation diameter of 5 cm and is therefore suitable for large HCCs. The effective ablation effect was demonstrated by the fact that 3 patients with small HCCs had complete ablation of tumors as shown by subsequent contrast computed tomography.

This study has shown that RFA is a safe and simple operative hemostasis method that can produce an excellent survival outcome. According to our treatment algorithm (Figure 1), TACE is the first treatment option since it can provide effective hemostasis with minimal invasiveness whereas RFA is the first-line operative approach when open hemostasis is required. In the study, RFA achieved effective hemostasis in a short period of ablation time. It sped up the whole operation procedure and, most importantly, it improved the overall hospital survival of patients who required open hemostasis.

In general, RFA is safe for and well tolerated by HCC patients. However, in patients with cirrhosis, the complication rate would be slightly higher. As found by one of our previous studies, a continuous ablation time of more than 36 min might induce intolerance in patients with cirrhosis [11]. Therefore, a long ablation time should be avoided.

Hemostasis of large ruptured HCCs is difficult even for RFA. For large lesions or when hemostasis could not be achieved in an initial 12-min cycle, the Pringle maneuver should be considered as a measure to decrease the blood flow to the lesion. In the study, a patient who had massive bleeding from the tumor underwent the Pringle maneuver for 60 min, and successful hemostasis was achieved. No patient in the study developed liver failure as a consequence of hemostasis by RFA. If RFA fails to stop tumor bleeding, hemostasis has to be performed by other surgical means such as hepatic artery ligation or liver resection, with an increased risk of hospital mortality.

Liver resection can be carried out for peripheral lesions in a very short period of time. With the advancement of laparoscopic surgery, new technology (e.g. LigaSure), new equipment (e.g. the Harmonic scalpel) and new linear staplers with good hemostatic effect are available [27], [28]. Theoretically these devices can speed up the resection of peripherally located lesions. Laparoscopic liver resection can be safely performed even in patients with cirrhosis [29]. Two patients in the study had liver resection for lesions located at the left lateral section with uneventful recovery.

The overall survival of HCC patients depends largely on the stage of disease [17], [30], [31]. Patients in both period 1 and period 2 presented with advanced tumors and cirrhosis. On multivariate analysis, patients with poor liver function had poorer survival. Their poor liver function precluded them from receiving further treatment such as locoregional therapy, targeted therapy, systemic therapy and hepatectomy, which could prolong their survival [32]–[36].

In conclusion, in the management of spontaneous rupture of HCCs, hemostasis by RFA instead of hepatic artery ligation can largely improve patients' hospital survival.
