# Correction: Management of Spontaneously Ruptured Hepatocellular Carcinomas in the Radiofrequency Ablation Era

**DOI:** 10.1371/journal.pone.0103921

**Published:** 2014-07-29

**Authors:** The PLOS ONE Staff

There are several errors in this article.

In the abstract and the Introduction section, the term “chemoembolization” should be “embolization”.

Throughout the article, including Figure 1, the abbreviation "TACE" should be "TAE", with the exception of the second sentence in the sixth paragraph of the Results section, row fifteen of Table 4, and the figure legend of Figure 3c.

**Figure 1:**
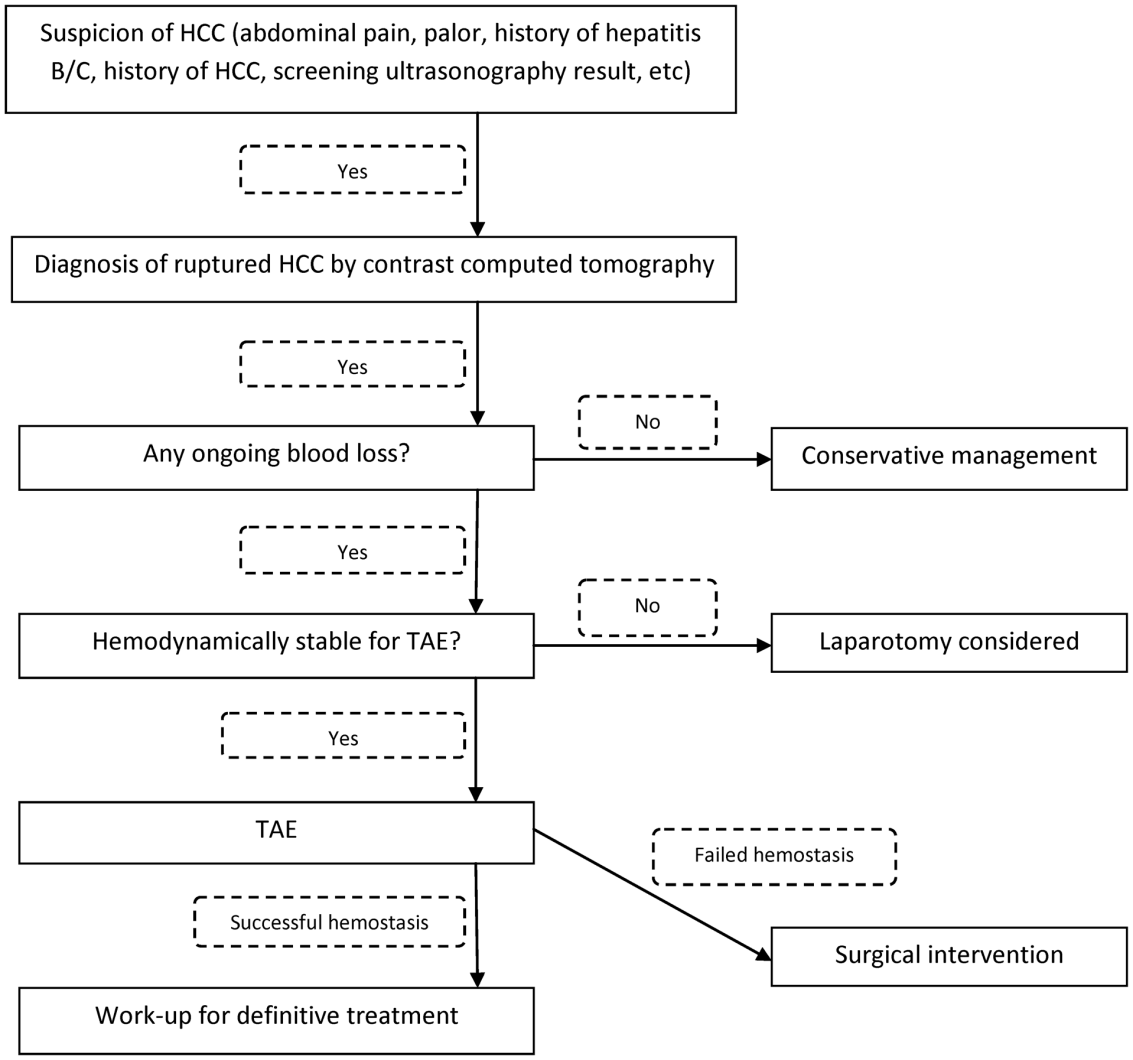
Treatment algorithm for spontaneous rupture of HCCs.

The correct version of the second sentence in the sixth paragraph of the Results section is: Sixteen patients in period 1 and 19 patients in period 2 received transarterial chemoembolization as a cancer treatment.

The correct version of Table 4 can be viewed below. The figure legend for Figure 3c is correct as is published.

**Table 4:** Factors that might affect patient survival.

	1991-2000			2001-2010			1991-2010		
	No. of patients	Median survival (months)	P	No. of patients	Median survival (months)	P	No. of patients	Median survival (months)	P
Tumor size (cm)			0.003			0.001			<0.0001
≤10	31	4.63		62	5.06		93	5.06	
>10	34	1.25		56	1.41		90	1.35	
Hemostasis by RFA			-			0.089			0.083
No	70	1.61		100	3.16		170	2.10	
Yes	0	-		19	6.61		19	6.61	
Hemostasis by surgery			0.588			0.870			0.653
No	52	1.84		106	3.68		158	2.33	
Yes	18	0.59		13	3.88		31	3.29	
Hemostasis by TAE			0.003			0.133			0.001
No	44	1.08		70	2.89		114	1.71	
Yes	26	6.54		49	4.86		75	5.06	
Subsequent TACE			0.068			0.517			0.120
Yes	16	4.63		19	5.16		35	5.16	
No	54	1.05		100	3.02		154	1.61	
Subsequent surgery			0.001			<0.0001			<0.0001
Yes	12	16.69		16	17.12		28	19.45	
No	58	1.22		103	2.56		161	1.61	
Shock on presentation			0.097			0.149			0.96
No	37	2.14		68	3.68		105	3.02	
Yes	33	0.59		51	4.83		84	1.38	
Child-Pugh class			<0.0001			0.197			<0.0001
A	30	8.18		55	4.86		85	5.75	
B	26	0.79		48	2.76		74	1.25	
C	9	0.36		16	0.62		25	0.62	
MELD score			0.0004			0.399			0.123
≤11	28	7.76		61	3.85		89	4.04	
>11	33	1.22		57	2.76		90	1.31	
Total bilirubin (umol/L)			0.0002			0.001			<0.0001
≤19	32	6.54		62	5.52		94	5.75	
>19	36	1.05		57	1.35		93	1.22	
Platelet (×10^9^/L)			0.679			0.168			0.449
≤192.5	41	1.35		53	5.06		94	3.85	
>192.5	27	1.94		66	2.56		93	2.17	
International normalized ratio			0.001			0.621			0.019
≤1.2	37	2.53		77	4.47		114	4.04	
>1.2	25	0.89		41	1.51		66	1.22	
Albumin (g/L)			0.063			0.443			0.086
≤34	35	1.08		67	2.89		102	1.31	
>34	33	4.63		52	4.24		85	4.47	

TACE, transarterial chemoembolization

MELD score, Model for End-stage Liver Disease score (to the nearest integer of the median)

The authors have provided corrected version of Figure 1 and Table 4, which can be viewed here.
